# Systemic immune-inflammation index as a prognostic biomarker to predict overall survival after primary stereotactic radiosurgery for brain metastases

**DOI:** 10.1007/s11060-025-05297-2

**Published:** 2025-10-15

**Authors:** Sukwoo Hong, Hirokazu Takami, Motoyuki Umekawa, Yuki Shinya, Hirotaka Hasegawa, Mariko Kawashima, Yosuke Kitagawa, Masashi Nomura, Shunsaku Takayanagi, Shota Tanaka, Nobuhito Saito

**Affiliations:** 1 Department of Neurosurgery, The University of Tokyo Hospital, 7-3-1 Hongo, Bunkyo-ku, Tokyo, 113-8655 Japan; 2 Department of Neurological Surgery, Mayo Clinic, Phoenix, AZ USA; 3 Department of Neurosurgery, University of Virginia, Charlottesville, VA USA; 4 Department of Neurosurgery, Saitama Medical Center, Saitama, Japan; 5 Gamma Knife Center, NTT Medical Center Tokyo, Tokyo, Japan; 6 Department of Neuro-Oncology, International Medical Center, Saitama Medical University, Saitama, Japan; 7 Department of Neurological Surgery, Okayama Graduate School of Medicine, Dentistry, and Pharmaceutical Sciences, Okayama, Japan

**Keywords:** SII, SIRI, Inflammation, Index, Radiosurgery

## Abstract

**Purpose:**

To evaluate the usefulness of the systemic immune-inflammation index (SII) and systemic inflammation response index (SIRI) in predicting local tumor control (LC) duration and overall survival (OS) in patients with brain metastases treated with primary stereotactic radiosurgery (SRS).

**Methods:**

All consecutive patients who underwent fractionated SRS between April 2018 and December 2022 were retrospectively analyzed. Statistical analyses included Cox regression and Kaplan-Meier curves.

**Results:**

A total of 132 metastases in 65 patients were analyzed. The median SII was 909 (IQR 524–1696), and the median SIRI was 1.6 (IQR 0.7–3.1). The median LC duration was 10 months, with 66.7% of lesions under control, 13.6% progressed, and 19.7% had unknown status due to insufficient imaging. The median OS was 11 months; 83% of patients had died by the last follow-up. SII and SIRI were not associated with LC duration in univariable Cox regression analysis. However, higher SII (*p* = 0.05) was significantly associated with worse OS in multivariable Cox regression analysis, along with male sex (*p* < 0.01) and gastrointestinal cancer origin (*p* = 0.02). These findings were also supported by Kaplan-Meier curves with log-rank tests. The optimal SII cutoff for predicting OS was 2600 (concordance index 0.72; hazard ratio 2.65). SIRI was associated with OS in univariate analysis (*p* = 0.03), but not multivariable analysis.

**Conclusion:**

This study suggests that higher SII was associated with worse OS and that it can be useful markers to predict OS, but not LC duration.

## Introduction

Brain metastases are the most common type of brain tumors in adults [1, 2]. The primary treatment options for local control include surgical resection and radiation therapies, such as stereotactic radiosurgery (SRS) [3]. Accurate prognosis prediction is critical for guiding clinical decision-making in patients with brain metastases. The diagnosis-specific graded prognostic assessment (DS-GPA) is one widely used system designed for patients with newly diagnosed brain metastases [4]. While DS-GPA is clinically valuable, it has limitations, including subjectivity, inter-rater variability, and the absence of objective laboratory markers. For example, it incorporates Karnofsky Performance Status, a subjective measure that can differ between evaluators. Similarly, extracranial metastasis status is subject to detection bias, which may also affect the accuracy of prognosis.

Systemic immune-inflammation index (SII) and systemic inflammation response index (SIRI)—both derived from routine complete blood count (CBC) with differential— were originally developed in hepatocellular and pancreatic cancer cohorts, respectively, and incorporate neutrophils, monocytes, platelets, and lymphocytes to reflect systemic inflammation and immune suppression. Higher values are consistently linked to worse prognosis, possibly indicating a tumor-promoting immune environment and impaired surveillance [5, 6]. Recently, SII and SIRI have been reported as useful prognostic markers in various cancers including lung, gastric, renal, and colorectal malignancies [7–12]. Since DS-GPA does not incorporate blood-based biomarkers, evaluating the prognostic value of SII and SIRI could provide additional clinical utility. The utility of these indices for brain metastases treated with surgical resection has been reported recently [13, 14]. However, few studies have investigated their role in patients undergoing SRS for brain metastases. Previous studies focused on other blood markers; therefore, it is important to evaluate SII and SIRI in patients receiving SRS for various types of brain metastases [15, 16]. 

This study aimed to evaluate whether SII and SIRI are useful for predicting overall survival (OS) and local tumor control (LC) duration after primary SRS.

## Methods

This study was approved by the Institutional Review Board (#2231, G10228) of our institution. Written informed consent for participation in the study was obtained from all patients. This study was designed in compliance with the Strengthening the Reporting of Observational Studies in Epidemiology statement [17]. 

### Patient selection and evaluation

**Fig. 1:**
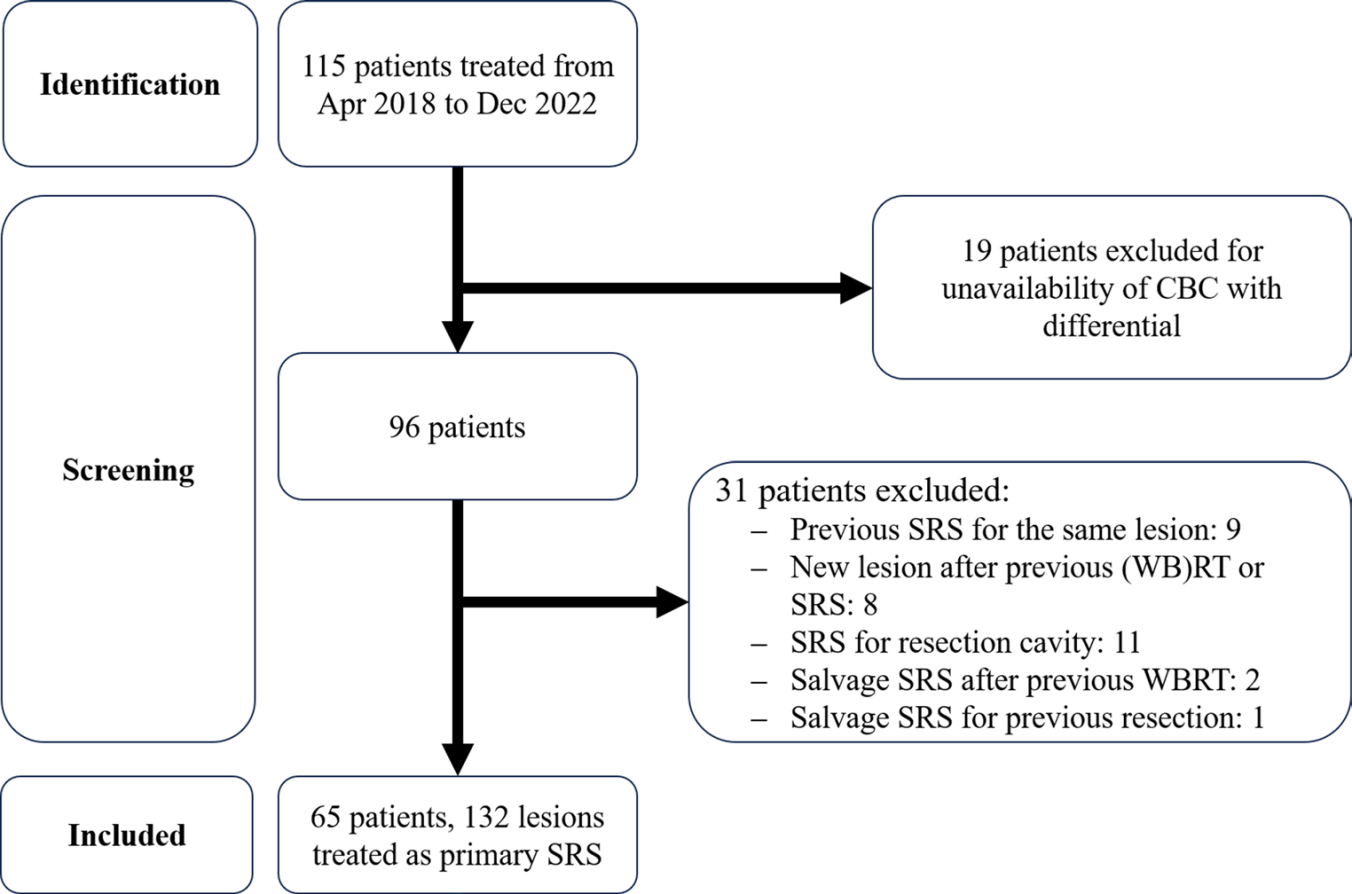
Flowchart showing patient selection for primary fractionated stereotactic radiosurgery (SRS). Abbreviations: CBC = complete blood count; (WB)RT = (whole-brain) radiation therapy

The institutional GammaKnife database was queried for patients who underwent fractionated SRS between April 2018 and December 2022 (Fig. 1). Patients were excluded based on the following criteria: (1) lack of adequate pre-SRS CBC with differential, (2) re-SRS for a lesion previously treated with SRS, (3) SRS for a new lesion in a patient who had received prior (whole-brain) radiation therapy or SRS, (4) adjuvant SRS for a resection cavity, and (5) salvage SRS for a lesion previously treated with surgical resection. No additional exclusions were applied based on age, performance status, or presence of leptomeningeal disease.

The most recent CBC prior to SRS was collected, and the neutrophil-to-lymphocyte ratio (NLR), platelet-to-lymphocyte ratio (PLR), and lymphocyte-to-monocyte ratio (LMR) were calculated. At the time of CBC measurement, none of the patients were receiving systemic cancer treatment or had active clinical evidence of infection or inflammation.

SII and SIRI were calculated as follows [5, 6]:

$$\:SII=(neutrophil\:count\times\:platelet\:count)/\left(lymphocyte\:count\right)$$

$$\:SIRI=(neutrophil\:count\times\:monocyte\:count)/\left(lymphocyte\:count\right)$$

Tumor volumes were calculated using Leksell GammaPlan^®^ (Elekta Instruments) based on gadolinium-enhanced magnetic resonance imaging (Gd-MRI).

### Radiosurgical procedure

SRS procedures and techniques were performed as previously described [18]. Typically, a 1-mm tumor margin was outlined with a 50% isodose line, prescribing a dose of 30 Gy in 3 fractions, 32 Gy in 5 fractions, and 38.5 Gy in 8 fractions [19]. All radiosurgical treatments were planned and approved by dedicated neurosurgeons and radiation oncologists involved in the procedure.

### Follow-up and outcome measurement

The primary outcome was OS, and the secondary outcome was LC duration, which was analyzed in an exploratory manner due to the limited number of events (i.e., failures of local control). Tumor progression was defined as a ≥ 20% increase in tumor volume according to the Response Assessment in Neuro-Oncology Brain Metastases (RANO-BM) criteria [20]. LC duration was calculated from the end date of SRS to the earliest of the following: (1) radiological evidence of tumor progression (event), (2) surgical resection due to radiation necrosis around SRS site (censor), or (3) the latest date of MRI or enhanced CT (if MRI was not feasible), which was considered a censoring point. OS was calculated from the end date of SRS to the date of the following: (1) death or (2) the latest follow-up date. All surviving patients were followed for at least 24 months after SRS, and deceased patients were followed until the time of death.

### Statistical analysis

To address potential sampling bias, continuous variables were treated as nonparametric and expressed as median values with interquartile ranges (IQR). Categorical variables were expressed as frequencies and percentages. Chi-square, Fisher’s exact, Mann-Whitney U tests (with or without Bonferroni correction), and Kruskal-Wallis tests were used as appropriate. Cox regression analysis was performed to evaluate associations between (pre-)treatment variables and tumor progression or death. Hazard ratios (HRs) with 95% confidence intervals (CIs) were calculated. Missing data were not imputed. For LC duration analyses, lesions lacking adequate post-SRS imaging to define either progression or a censoring time were excluded a priori (no LC duration recorded). The proportional hazards assumption for all covariates was assessed using Schoenfeld residuals, and no violations were observed [21]. 

Variables included in the multivariable analysis were selected using the forward likelihood ratio stepwise method, with a p-value cutoff of 0.25. Multicollinearity was evaluated using a correlation coefficient cutoff of 0.7 (Pearson’s r, Spearman’s rho, or Kendall’s tau) and variance inflation factor cutoff of 5. An events-per-variable of at least 10 was maintained. To dichotomize continuous variables, candidate thresholds were scanned from the 10th to 90th percentiles of the observed values in 1-percentile increments. At each candidate cutoff, the cohort was split into “high” and “low” groups, and a multivariable Cox proportional hazards model (including the same covariates as above) was fit. The discriminative ability of each model was quantified by Harrell’s concordance index (C-index), and the threshold yielding the highest C-index was selected as the optimal cutoff. Kaplan-Meier curves and the log-rank test were used to evaluate OS using significant features from the multivariable analysis and dichotomized continuous variables. p-values ≤ 0.05 were considered significant. Statistical analyses were performed using SPSS (version 25.0, IBM Corp.) and Python.

## Results

### Patient characteristics and treatments

A total of 132 metastases were identified in 65 patients. Baseline patient and metastasis characteristics are summarized in Table 1. Primary cancer types per patient included lung (43%), breast (11%), gastrointestinal (22%), and renal (8%) cancers. Thirty-eight patients (59%) received primary SRS for two or more brain metastases. The median SII was 909 (IQR 524–1696), and the median SIRI was 1.6 (IQR 0.7–3.1). None of the patients had an active infection, and 22% were receiving steroids at the time of blood testing. The median interval between the blood test and SRS was 6 days (IQR 1–15). There was no strong correlation between the interval and SII (Spearman’s ρ = − 0.25) or between the interval and SIRI (Spearman’s ρ = − 0.31). The median tumor volume was 3.2 mL (IQR 0.8–8.5), and the median sum tumor volume per patient was 9.3 mL (IQR 4.8–16.6).

**Table 1:** Baseline characteristics per patient

*N* (patients)	65
Age (yrs)	71 (IQR 60–76)
Male	36 (55%)
ECOG performance status	
0 1 2 3	20 (31%) 11 (17%) 20 (31%) 14 (22%)
Cancer origins	
Lung Breast Gastrointestinal Renal Etc	28 (43%) 7 (11%) 14 (22%) 5 (8%) 11 (16%)
Number of metastases	
1 ≥ 2	27 (42%) 38 (59%)
Sum of longest tumor diameter (mm)	41 (IQR 27–53)
Sum of all tumor volume (mL)	9.3 (IQR 4.8–16.6)
Neutrophil (/mcL)	5,000 (IQR 3,700–7,100)
Lymphocyte (/mcL)	1,300 (IQR 910–1,900)
Monocyte (/mcL)	400 (IQR 300–600)
Platelets (/mcL)	249,000 (IQR 205,000–323,000)
NLR	3.84 (IQR 2.25–6.00)
PLR	197 (IQR 113–293)
LMR	3.00 (IQR 2.08–5.21)
SII	909 (IQR 524–1696)
SIRI	1.6 (IQR 0.7–3.1)
On steroid at the time of blood test	14 (22%)

ECOG = Eastern Cooperative Oncology Group; LMR = lymphocyte-monocyte ratio; NLR = neutrophil-lymphocyte ratio; PLR = platelet-lymphocyte ratio; SII = systemic immune-inflammation index; SIRI = systemic inflammation response index

SRS treatment parameters are as follows. Among all lesions, 50% were treated with 3-fraction SRS, for which the median marginal dose was 30 Gy (IQR 30–30) and the median central dose was 60 Gy (IQR 50–60). For 5-fraction or 8-fraction SRS (48%), the median marginal dose was 32 Gy (IQR 32–35), and the median central dose was 64 Gy (IQR 51–70).

### LC duration and OS

**Fig. 2:**
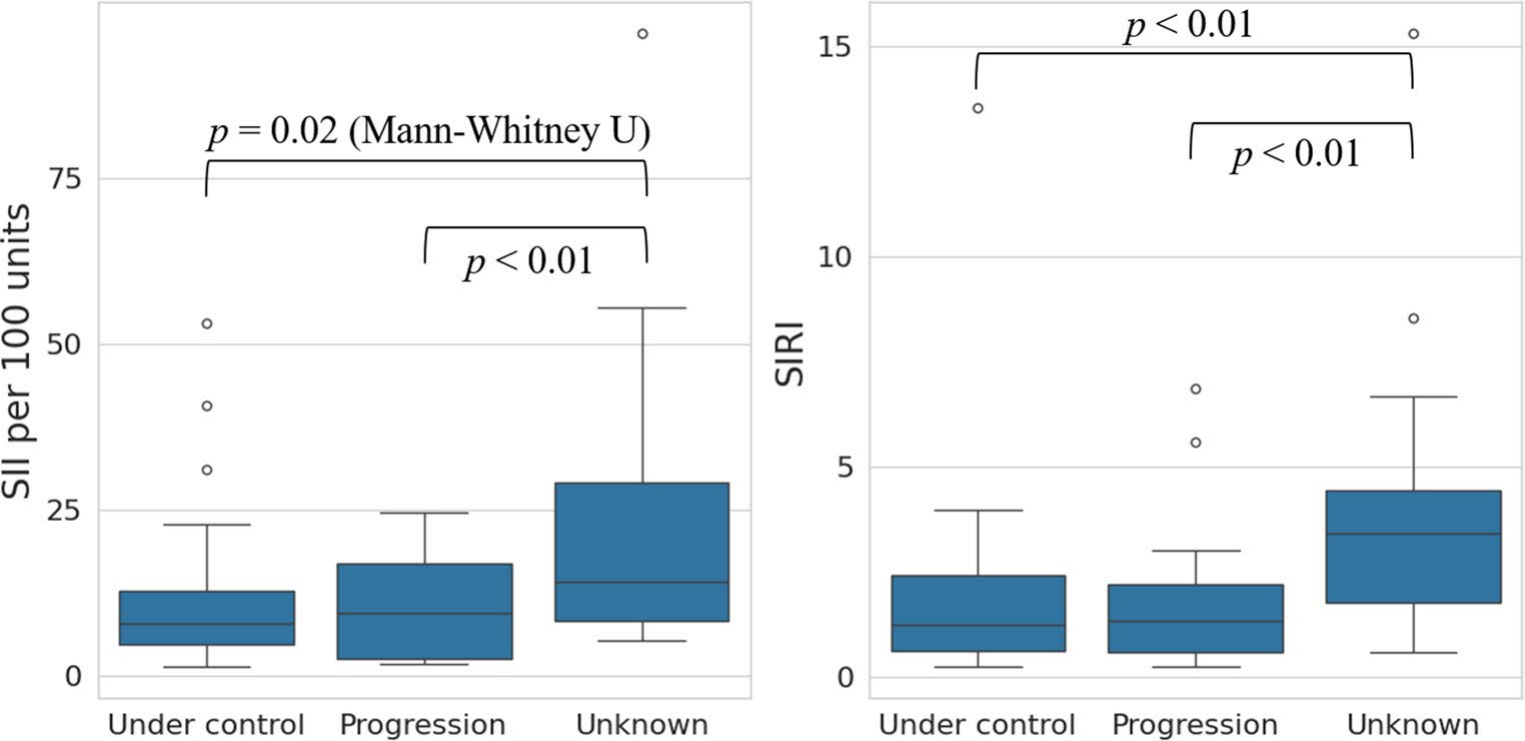
Systemic immune-inflammation index (SII) and systemic inflammation response index (SIRI) by tumor control status. Metastases with unknown tumor control had significantly higher SII and SIRI than those with controlled or progressive disease

Of the 132 metastases, 88 (66.7%) remained controlled (at the last follow-up imaging), 18 (13.6%) progressed, and 26 (19.7%) had unknown outcomes due to insufficient MRI follow-up for medicosocial reasons. The SII and SIRI values were significantly higher in lesions with unknown outcomes compared to those with known outcomes (controlled or progressed), suggesting their potential associations with early loss to follow-up or worse prognosis possibly due to systemic disease progression (Fig. 2). Patients with unknown LC status had significantly shorter OS (median 1.0 months, IQR 1.0–2.8) compared with those with known status (median 18.0 months, IQR 7.5–35.0; *p* < 0.01). The median LC duration was 10 months (IQR 4–19). In univariable Cox regression analysis for tumor progression, neither SII nor SIRI was associated with progression risk. Among all baseline characteristics, only gastrointestinal cancer origin (reference: lung cancer) was significantly associated with worse LC duration (HR 8.49, 95% CI 2.19–32.91; *p* < 0.01). The sample size of progression, 18/132, precluded a multivariable analysis from the perspective of events-per-variable. Sensitivity analyses were performed to assess the potential impact of unknown progression status. In the best-case scenario, treating unknowns as censored at last follow-up, SII and SIRI were not significantly associated with LC duration (HR 0.96, *p* = 0.17; HR 0.82, *p* = 0.20, respectively). In the worst-case scenario, treating unknowns as progression events, both SII and SIRI were significantly associated with LC duration (HR 1.03, *p* < 0.01; HR 1.23, *p* < 0.01, respectively).

The median OS was 11 months (IQR 3–28). At the time of the latest follow-up, 54 patients (83%) had died. Of all deaths, 40 (61.5%) were cancer-related, 8 (12.3%) were central nervous system-related, and 6 (9.2%) were due to other causes. In univariable analysis for death (Table 2), male sex (HR 2.63, 95% CI 1.46–4.71, *p* < 0.01), gastrointestinal cancer origin (HR 2.49, 95% CI 1.24–5.03, *p* = 0.01), higher SII for 100 units (HR 1.02, 95% CI 1.00–1.04, *p* = 0.04), and higher SIRI (HR 1.12, 95% CI 1.01–1.25, *p* = 0.03) were significantly associated with worse OS. In multivariable analysis, male sex, gastrointestinal cancer origin, and higher SII per 100 units (HR 1.02, 95% CI 1.00–1.04, *p* = 0.05) remained independently associated with increased risk of death. Of note, since SII and SIRI were highly correlated (Pearson’s *r* = 0.85), two separate multivariable models were created. In the model including SIRI, it was not statistically significant.

**Table 2:** Cox regression analysis for overall survival after SRS

	Univariate		Multivariable	
	HR (95% CI)	p-value	HR (95% CI)	p-value
Age	1.01 (0.99–1.03)	0.47	/	/
Male	2.63 (1.46–4.71)	**0.00**	2.88 (1.52–5.48)	**< 0.01**
Cancer origin (vs. Lung) Gastrointestinal Etc	2.49 (1.24–5.03) 1.46 (0.78–2.74)	0.04 **0.01** 0.23	2.36 (1.16–4.81) 1.94 (1.00–3.77)	0.03 **0.02** **0.05**
Number of metastases (continuous)	1.32 (0.96–1.82)	0.09	1.34 (0.97–1.86)	0.08
Sum longest diameter (mm)	1.01 (1.00–1.03)	0.20	/	/
Sum volume (mL)	1.01 (0.99–1.03)	0.20	/	/
NLR (continuous)	1.05 (0.97–1.14)	0.22	/	/
PLR (per 10 units, continuous)	1.01 (1.00–1.03)	0.12	/	/
LMR (continuous)	0.88 (0.77–1.01)	0.07	/	/
SII (per 100 units, continuous)	1.02 (1.00–1.04)	**0.04**	1.02 (1.00–1.04)	**0.05**
SIRI (continuous)	1.12 (1.01–1.25)	**0.03**	/	/
On steroid at the time of blood test	1.14 (0.57–2.29)	0.71	/	/

LMR = lymphocyte-monocyte ratio; NLR = neutrophil-lymphocyte ratio; PLR = platelet-lymphocyte ratio; SII = systemic immune-inflammation index; SIRI = systemic inflammation response index

### Exploration of SII and SIRI cutoff values for OS

**Fig. 3:**
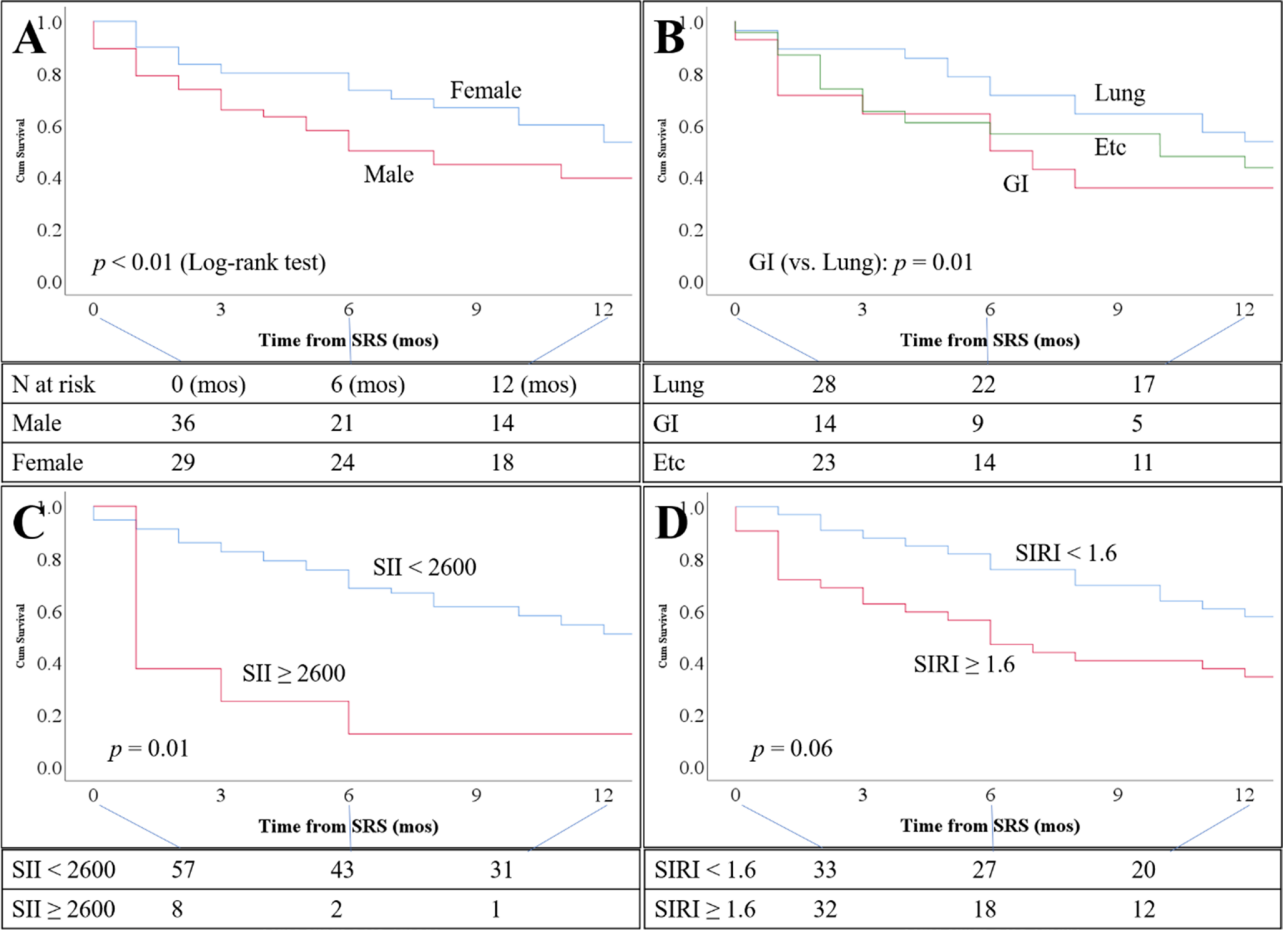
Kaplan-Meier curves of overall survival (OS). **A**: Male patients had worse OS than females. **B**: Gastrointestinal (GI) cancer had worse OS than lung cancer origin. **C**: Systemic immune-inflammation index (SII) ≥ 2600 was associated with worse OS. **D**: Systemic inflammation response index (SIRI) ≥ 1.6 tended to be associated with worse OS

To dichotomize SII, we tested cutoff values across the 10th–90th percentile range using a multivariable Cox model (including sex, cancer origin, and SII) and selected those with the highest C-index. Among them, 2583 gave the highest C-index (0.724), but for clinical interpretability, we selected 2600 as the optimal cutoff. This value yielded an HR of 2.65 (95% CI 1.17–5.99; *p* = 0.02) for OS. Kaplan–Meier survival curves stratified by key variables are shown in Fig. 3.

For comparison, the optimal cutoffs for NLR, PLR, LMR, and SIRI were 1.80 (C-index 0.693), 271 (C-index 0.694), 3.68 (C-index 0.687), and 1.6 (C-index 0.698), respectively.

### Exploratory subgroup analyses for OS

Patients receiving steroids had significantly higher SII (median 1585, IQR 818–4153) compared with those not on steroids (median 784, IQR 451–1481; Mann–Whitney U, *p* < 0.01). Similarly, SIRI was higher in patients on steroids (median 3.71, IQR 1.34–7.29) compared with those not on steroids (median 1.28, IQR 0.59–2.60; Mann–Whitney U, *p* < 0.01). Among patients receiving steroids (*n* = 14, deaths = 10), none of the variables listed in Table 2 were statistically significant in univariate Cox regression analysis. In patients not receiving steroids (*n* = 51, deaths = 44), male sex (HR 2.48, *p* < 0.01), number of metastases (HR 1.12, *p* = 0.01), and SII per 100 units (HR 1.05, *p* = 0.02) remained significant in multivariable Cox regression analysis. The optimal SII cutoff was 1848 (C-index 0.716). Among patients with lung cancer (*n* = 28), male sex (HR 4.88, *p* = 0.01), number of metastases (HR 1.96, *p* = 0.04), and SIRI (HR 1.25, *p* = 0.02) were statistically significant in univariate analysis. In multivariable analysis, only male sex (HR 2.69, *p* < 0.01) remained significant. Among patients with gastrointestinal cancer (*n* = 14), none of the variables listed in Table 2 reached statistical significance in univariate analysis. Number of metastases (HR 2.04, *p* = 0.10) and SIRI (HR 1.32, *p* = 0.07) showed a trend toward association.

## Discussion

This study assessed the prognostic utility of the SII and SIRI in patients undergoing fractionated SRS for brain metastases. While neither index was associated with LC duration, a higher SII was significantly associated with OS. In a multivariable model including sex, cancer origin, and number of metastases, an SII cutoff of 2600 demonstrated an acceptable C-index of 0.724. To the best of our knowledge, this is the first study to identify SII as a significant prognostic factor for OS in patients with brain metastases treated with primary SRS. A previous similar study on non-small cell lung cancer found that a higher SIRI was independently associated with shorter OS in multivariable Cox regression, along with the NLR and LMR [8]. In contrast, in our study, neither NLR, PLR, nor LMR was statistically significant in univariable analysis. and SIRI did not remain significant in multivariable analysis. These discrepancies may be due to the heterogeneity of patient populations and the smaller sample size in our study. Therefore, further studies with larger cohorts are warranted. In subgroup analyses by primary tumor origin, SII was no longer statistically significant, with male sex remaining significant in the lung cancer subgroup and no variables reaching significance in the gastrointestinal cancer subgroup. These results suggest that the prognostic value of SII may vary across tumor types; however, limited subgroup sizes reduce statistical power, so these findings are exploratory and hypothesis-generating.

The HR for SII per 100 units was statistically significant (HR 1.02), though its clinical significance may be limited. However, using an SII cutoff of 2600, we observed a HR of 2.65, which was both statistically and clinically meaningful. The cutoff of 2600 is higher than in previous studies, which reported 594 (surgical resection cohort, excluded in the current study) and 1095 (cohort of exclusively non-small cell lung cancer) [8, 14]. This difference may reflect variations in the patient population and study methodology. Although a SII cutoff of 2600 was statistically significant, only eight patients met this threshold (SII ≥ 2600), whereas 32 patients had SIRI ≥ 1.6. Therefore, the SIRI cutoff may be more clinically useful despite the stronger statistical signal of SII. Unlike the prior study, we included patients who were receiving corticosteroids at the time of CBC. Given that corticosteroid use is common in this population to manage peritumoral edema and neurological symptoms, we chose to include these patients to better reflect real-world clinical settings. Admittedly, in the subgroup of patients receiving steroids, SII was not a statistically significant factor for OS. This suggests that the prognostic utility of SII may be reduced in patients on steroids, and further studies with larger cohorts are needed to verify this finding.

A previous study involving 250 patients reported that total brain metastasis volume was significantly associated with OS [22]. However, our study did not find an association between sum tumor volume and OS, which may again be attributable to the smaller sample size (*n* = 65). Similarly, another study of 52 patients with esophageal cancer also found that cumulative intracranial tumor volume was not associated with OS [23]. Previous studies identified male sex as a poor prognostic factor for OS. One multivariable Cox regression analysis of 190 patients with brain metastases found that male sex, age ≥ 65, more than three brain metastases, and absence of molecular targeted therapy were independently associated with shorter OS [24]. Another study also reported an association between male sex and poorer OS outcomes [25]. According to the American Cancer Society, the 5-year relative survival rates for distant-stage gastrointestinal cancers are 5% for esophageal, 7% for gastric, and 13% for colorectal cancers—generally lower than those for distant-stage lung cancers, which are 12% for non-small cell and 4% for small cell lung cancer [26–29]. In contrast, distant-stage breast cancer has a 5-year relative survival rate of 32%, which is higher than that of the aforementioned cancers [30]. These findings support our results, which showed that male sex and gastrointestinal cancer origin were independently associated with worse OS.

Our study did not show SII and SIRI to be associated with LC duration. One possible explanation is that these indices reflect systemic inflammatory status, which may be more strongly linked to OS rather than local progression following SRS. In contrast, LC duration may be less influenced by systemic inflammation. Another possible explanation is that progression of brain metastases may be less likely to manifest systemic inflammatory changes due to the presence of the blood–brain barrier. A recent meta-analysis revealed poor correlation between peripheral (e.g., interferon, tumor necrosis factor) and central inflammatory markers [31], suggesting that systemic inflammatory status may not accurately reflect CNS inflammation. Studies specifically in patients with brain metastases appear to be limited. Additionally, 26 lesions (19.6%) had unknown LC status due to lack of imaging follow-up, often for medicosocial reasons, despite all patients being clinically followed for at least 24 months. Notably, lesions with unknown LC had significantly higher SII and SIRI, raising the possibility that incomplete follow-up may have biased the LC duration analysis and potentially masked a true association. These lesions also had shorter OS compared with those with known LC, suggesting that the unknown LC status was likely related to patients’ poor condition rather than to systematic imaging follow-up. Sensitivity analyses showed that the relationship between SII/SIRI and LC duration was highly dependent on how these missing outcomes were handled, precluding definitive conclusions about their prognostic value for LC duration. Finally, as tumor progression occurred in only 18 out of 132 tumors, multivariable Cox regression was not performed. For future studies, evaluation in larger cohorts is warranted, and SII could be incorporated into existing prognostic tools such as DS-GPA, or combined with molecular and genetic biomarkers, to improve risk stratification and facilitate more individualized, clinically meaningful predictions.

This study has several limitations. First, its retrospective design and small sample size may limit generalizability. However, because the events-per-variable ratio was maintained above 10, the risk of overfitting in the OS models is likely small. Second, the cohort was heterogeneous in terms of primary tumor origin and systemic treatment backgrounds. Additionally, 19 patients were excluded due to missing CBC with differential data, which could have introduced bias. Future validation in larger, prospective studies or systematic reviews with meta-analyses is warranted to confirm these findings. Third, related to the second limitation, we did not account for whether patients had extracranial metastases, primary tumor control, or received systemic treatments such as molecular targeted therapies or immune checkpoint inhibitors, all of which may affect OS. These factors might have confounded the results.

## Conclusion

Elevated SII is associated with poorer OS after primary SRS for brain metastases. CBC with differential, a widely available and cost-effective test, may provide useful prognostic information in clinical decision-making for these patients.

## Data Availability

The datasets generated during and/or analyzed during the current study are available from the corresponding author(s) on reasonable request.
